# The association of Serratus Anterior Plane blocks with postoperative opioid use and analgesia following simple lumpectomy: a retrospective cohort analysis

**DOI:** 10.1186/s12871-022-01834-y

**Published:** 2022-09-15

**Authors:** Brenton Alexander, Soraya Mehdipour, Seung Woo Lee, Engy T. Said, Rodney A. Gabriel

**Affiliations:** 1 Department of Anesthesiology, University of California, 9400 Campus Point Dr, La Jolla, San Diego, California 92037 USA; 2 Division of Biomedical Informatics, Department of Medicine, University of California, La Jolla, San Diego, California USA; 3 Division of Perioperative Informatics, Department of Anesthesiology, University of California, La Jolla, San Diego, California USA

**Keywords:** Serratus anterior plane, Regional anesthesiology, Lumpectomy, Breast surgery, Enhanced recovery after surgery

## Abstract

**Background:**

The potential benefit of regional interventions for simple lumpectomy breast cancer surgeries has not been well investigated. Understanding which patients to *not* offer a regional intervention to can be just as important as knowing which would benefit. It is unclear whether fascial plane blocks, such as serratus anterior plane (SAP) block, should be routinely performed for less extensive breast surgeries. Therefore, our goal in this retrospective cohort study was to evaluate the association of integrating SAP blocks into a standard perioperative multimodal analgesia plan in patients undergoing simple lumpectomies (without node biopsies) with perioperative opioid consumption. As secondary outcomes, we also analyzed postoperative pain scores and post-anesthesia care unit (PACU) length of stay.

**Methods:**

This was a single institution retrospective cohort study (surgical site infiltration only versus SAP block cohorts) assessing the association of SAP blocks to our outcomes of interest. In the adjusted analysis, we created matched cohorts using 1:1 (surgical site infiltration only: SAP block) propensity-score matching using nearest neighbor-matching without replacement. To compare the primary and secondary outcomes in the matched cohorts, we used the Wilcoxon signed rank test. A *P*-value of < 0.05 was considered statistically significant.

**Results:**

There were 419 patients included in the analysis, in which 116 (27.7%) received a SAP block preoperatively in addition to our standard perioperative analgesia plan. In an unadjusted analysis, no differences were seen in perioperative opioid consumption, PACU pain scores, and PACU length of stay. Among the matched cohorts, the median [quartile] perioperative opioid consumption in the surgical site infiltration only versus SAP block cohorts were 10 mg [10, 13.25 mg] and 10 mg [7, 15 mg], respectively (*P* = 0.16). No differences were seen in the other outcomes.

**Conclusions:**

In this study, we evaluated the impact of SAP blocks on patients undergoing simple lumpectomies, which are relatively less involved breast surgeries. We concluded that routine use of preoperative regional anesthesia is not beneficial for these specific patients. Future studies should focus on identifying patients that would directly benefit from regional interventions.

## Background

Breast surgery encompasses a heterogeneous group of procedures that vary in degree of invasiveness, sensory innervation, and risk of acute and chronic pain [1–6]. This makes interpretation of data from studies evaluating these patients’ perioperative pain management challenging as many studies will group all types (or partial subsets) of breast surgery together. Specific details of each patient’s individual surgical intervention are very important for evaluating and implementing potential pain treatment approaches. For example, the decision to include a regional anesthetic in a breast surgical patient’s perioperative pain plan will be very different for a chronic pain patient undergoing a mastectomy when compared to an opioid-naive patient undergoing a simple lumpectomy. Mastectomies with or without axillary lymph node dissections are at an elevated risk of acute and chronic pain and would therefore likely benefit from a regional intervention [3, 5–7], especially as poor acute pain management has been linked to the development of chronic breast pain [1, 6, 8]. However, the potential benefit of regional interventions for less invasive breast cancer surgeries has not been well investigated. Knowing which patients to *not* offer a regional intervention can be just as important as knowing which would benefit, especially as additional regional procedures can be resource consuming, uncomfortable for patients, and carry a small risk of pneumothorax [9], nerve damage and intravascular injection.

When discussing the specific regional procedure being used, truncal fascial plane blocks [including pectoral nerves (PECS), Erector Spinae Plane (ESP), and Serratus Anterior Plane (SAP)] have gained popularity as an alternative to paravertebral (PV) and central neuraxial blockade as they may be less technically challenging and have lower side effect profiles [10]. While there is no clear evidence of increased risks associated with paravertebral and epidural access, our institution has seen a higher rate of hemodynamic and pleural complications with PV when compared to more distal blocks. Of these, SAP is a relatively newer interfacial approach for perioperative pain control in patients undergoing surgery involving the chest wall [10, 11]. The SAP block targets lateral cutaneous branches of intercostal nerves T2-T9 as they pass superficial or deep to the serratus anterior muscle in the lateral chest wall [12] and innervates the anterolateral chest wall. The efficacy of this block for breast surgery has been investigated, particularly when compared to paravertebral (PVB), PECS and ESP blocks [13–17].

It is unclear whether fascial plane blocks, such as SAP block, should be routinely performed for less extensive breast surgeries. Therefore, our goal in this retrospective cohort study was to evaluate the association of integrating SAP blocks into a standard perioperative multimodal analgesia plan in patients undergoing simple lumpectomies (without node biopsies) with perioperative opioid consumption. Thus, we compared two cohorts: SAP blocks (with surgical site infiltration) versus surgical site infiltration only. As secondary outcomes, we also analyzed postoperative pain scores and post-anesthesia care unit (PACU) length of stay. Patients in both cohorts received local anesthesia infiltration at the surgical site by the surgeon in addition to standard non-opioid and opioid medication administration.

## Methods

### Study sample

This retrospective study was approved by our institution’s (UC San Diego) Human Research Protections Program for the collection of data from our electronic medical record system and the informed consent requirement was waived. Data were manually collected retrospectively from the electronic medical record system by one clinician (S.M.) from the University of California, San Diego Healthcare System. The manuscript adheres to the applicable EQUATOR guidelines for observational studies.

Data from all patients that underwent simple lumpectomy from a three-year period during 2019–2021 at our outpatient surgery center were extracted. Cases that included a concomitant surgery were excluded. For the subset of breast surgical patients undergoing a simple lumpectomy, the procedure is typically a small excision of a wedge of subcutaneous breast tissue. Depending on whether the surgery is performed medial or lateral to the nipple, the anterior or lateral cutaneous branches of the intercostal nerves will contribute innervation to the operative area, respectively. All patients were given preoperative acetaminophen (unless contraindicated). Intraoperatively, patients underwent either monitored anesthesia care with natural airway or general anesthesia with supraglottic airway. Anesthesia was maintained via propofol infusion and/or volatile anesthetic. Intravenous fentanyl and hydromorphone were given at the discretion of the anesthesiologist. Upon surgical closure, for all patients, the surgeon infiltrated local anesthesia (bupivacaine 0.25%) into the surgical field. Postoperatively, patients received fentanyl, intravenous hydromorphone, or oxycodone as needed.

In 2021, our regional anesthesia team implemented the addition of preoperative SAP blocks. In the preoperative room, patients were placed in the lateral position (operative side up). Ultrasound-guidance placed in a coronal plane at the midaxillary line and rib 4, rib 5, latissimus dorsi, and serratus anterior muscle were identified. A 20-gage Tuohy needle was then inserted in-plane and directed towards rib 5. The needle was inserted caudad to the probe. Local anesthetic (ropivacaine or bupivacaine) with 1:400,000 of epinephrine was deposited deep or superficial to the serratus anterior muscle (ranging from 20 to 30 mL per side). Patients receiving the SAP block also received surgical site infiltration. Thus, patients from 2019 to 2020 were in the “surgical infiltration only cohort” and patients from 2021 were in the “SAP cohort”. No other significant changes occurred between the two cohorts with respect to surgical or anesthetic management.

### Primary objective and data collection

The primary outcome measurement was perioperative opioid consumption, which was defined as total opioid use intraoperatively and in the PACU – measured in intravenous morphine equivalents (MEQ). Secondary outcomes included median PACU pain scores (measured in numeric rating scale [NRS]), maximum PACU pain score (NRS), and PACU length of stay (minutes). Potential confounder variables that were collected included age, body mass index, American Society of Anesthesiologists score, history of anxiety, history of depression, history of chronic pain, breast mass size (measured in cm for largest dimension), whether surgery was bilateral, whether mass was located medial to the breast, and primary anesthesia type (general anesthesia versus monitored anesthesia care).

### Statistical analysis

All statistical analysis was performed using R (Version 3.6.1). We compared baseline characteristics and outcomes in the surgical site infiltration only and the SAP block cohorts. For continuous and categorical variables, we used Wilcoxon rank sum test and chi-square test, respectively, to assess statistically significant differences. In the unadjusted analysis, outcomes were compared using Wilcoxon rank sum test.

Next, we performed a multivariable linear regression modeling the use of SAP block with perioperative opioid consumption. In this model, we controlled for age, body mass index, American Society of Anesthesiologists score, depression, anxiety, chronic pain history, breast mass size, and bilateral surgery. We reported the estimates, standard errors, and *P*-values for each variable included in the model. A *P* < 0.05 was considered statistically significant.

To create matched cohorts, we performed 1:1 (surgical site infiltration only: SAP block) propensity-score matching using nearest neighbor-matching without replacement. For this, we set the caliper at 0.2 standard deviations of the logit of the estimated propensity score. The propensity score for each cohort was calculated using logistic regression based on all the confounders listed above. The covariates were included due to their theoretical association with postoperative pain. An absolute standardized mean difference less than or equal to 0.2 for each covariate was considered adequate for balanced matching. To compare the primary and secondary outcomes in the matched cohorts, we used the Wilcoxon signed rank test. A *P*-value of < 0.05 was considered statistically significant.

#### Power analysis

Historically – prior to initiation of SAP blocks for simple lumpectomies – the average perioperative opioid consumption [standard deviation] was 11.3 mg MEQ [5.9 mg]. If we assume clinical significance is reduction of opioids by 25%, we would require 65 patients in each cohort (with power of 0.8 and alpha = 0.05).

## Results

There were 419 patients included in the analysis, in which 116 (27.7%) received a SAP block preoperatively in addition to our standard perioperative analgesia plan. All patients received surgical site infiltration with local anesthesia. The two cohorts studied were those who received SAP blocks and surgical site infiltration versus surgical site infiltration only. There were no differences in age, body mass index, ASA score, depression, anxiety, chronic pain, breast mass size, surgery laterality, medial location of mass, or primary anesthesia type between the cohorts (Table 1). There were no major complications (local anesthetic toxicity, pneumothorax, major hemodynamic shifts) noted for any patient included in this study.

**Table 1:** Patient characteristics, surgical characteristics, and outcomes in the No Block versus Serratus Block cohorts for continuous variables, the median values were compared using Wilcoxon ranked sum test for categorical variables, chi-squared analysis was performed

	No Block		Serratus Block		
	No Block	%	n	%	***P***-value
Total	303		116		
*Patient Characteristics*					
Age (years)	49 [41, 61]		52 [43.8, 63]		0.09
Body Mass Index (kg/m2)	25.6 [22.3, 30.2]		27.1 [22.7, 30.9]		0.37
ASA score ≥ 3	45	14.9	21	18.1	0.51
Depression	36	11.9	21	18.1	0.14
Anxiety	36	11.9	13	11.2	0.96
Chronic Pain	127	41.9	7	6.0	0.52
*Surgical Characteristics*					
Breast Mass Size (largest dimension in cm), median [quartile]	12 [5, 24.5]		11 [6.8, 17]		0.32
Breast Mass Located Medial to Nipple	68	22.4	29	25.0	0.67
Bilateral Surgery	8	2.6	2	1.7	0.85
General Anesthesia (versus Monitored Anesthesia Care)	258	85.1	106	91.4	0.13
*Outcomes*					
Perioperative Opioid Consumption (MEQ mg), median [quartile]	10 [10, 14.5]		10 [7, 15]		0.33
Median PACU pain score (NRS), median [quartile]	1 [0, 4]		1.25 [0, 4]		0.81
Maximum PACU pain score (NRS), median [quartile]	3 [0, 5.5]		4 [0, 6]		0.11
PACU length of stay (minutes), median [quartile]	80 [62, 100]		78.5 [64, 96.25]		0.68

*Abbreviations*: *ASA* American Society of Anesthesiologists, *MEQ* intravenous morphine equivalents, *NRS* numeric rating scale, *PACU* post-anesthesia care unit

The median [quartile] perioperative opioid consumption in the surgical site infiltration only versus SAP block cohorts were 10 mg [10, 14.5 mg] and 10 mg [7, 15 mg], respectively (*P* = 0.33). The median [quartile] PACU pain score on the NRS scale in the surgical site infiltration only versus SAP block cohorts were 1 [0, 4] and 1.25 [0, 4], respectively (*P* = 0.81). The median [quartile] maximum PACU pain score on the NRS scale in the surgical site infiltration only versus SAP block cohorts were 3 [0, 5.5] and 4 [0, 6], respectively (*P* = 0.11). The median [quartile] PACU length of stay in the surgical site infiltration only versus SAP block cohorts were 80 minutes [62, 100 minutes] and 78.5 minutes [64, 96.25 minutes], respectively (*P* = 0.68).

Next, we performed a multivariable linear regression modeling the utilization of serratus block (versus surgical site infiltration only) to perioperative opioid consumption (Table 2).

**Table 2:** Multivariable linear regression modeling utilization of serratus block with perioperative opioid consumption. All listed variables were included in the model as confounders

Variable	Estimate	Standard Error	***P***-value
Serratus versus No Block	−0.73	0.62	0.24
Age (years)	−0.04	0.02	0.05
Body Mass Index (kg/m2)	0.21	0.05	< 0.001
ASA score ≥ 3	−0.19	0.85	0.83
Depression	−0.45	0.92	0.62
Anxiety	−0.11	0.95	0.89
Chronic Pain	0.76	1.39	0.59
Breast Mass Size (largest dimension in cm)	0.01	0.02	0.51
Breast Mass Medial to Nipple	−0.35	0.66	0.59
Bilateral Surgery	1.98	1.82	0.28
General Anesthesia	0.66	0.84	0.43

*Abbreviations*: *ASA* American Society of Anesthesiologists

The model controlled for age, body mass index, ASA score, depression, anxiety, chronic pain, breast mass size, bilateral surgery, medial location of the mass, and primary anesthesia type. There was no statistically significant association with SAP block to perioperative opioid consumption (estimate = − 0.73, standard error = 0.62, *P* = 0.24). There was an association with body mass index (kg/m2) to perioperative opioid consumption (estimate = 0.21, standard error = 0.05, *P* < 0.001).

We also created 1:1 propensity-matched cohorts controlling for age, body mass index, ASA score, depression, anxiety, chronic pain, breast mass size, bilateral surgery, medial location of mass, and primary anesthesia type. Between both cohorts, all variables were adequately matched based on an absolute standardized mean difference of less than 0.2 for all variables (Table 3).

**Table 3:** Comparison of No Block versus Serratus Block in the propensity-matched cohorts

	No Block		Serratus Block		
	No Block	%	n	%	SMD
Total	116		116		
*Patient Characteristics*					
Age (years)	53 [44, 64]		52 [43.8, 63]		0.09
Body Mass Index (kg/m2)	25.9 [23, 31]		27.1 [22.7, 30.9]		0.06
ASA score ≥ 3	22	19.0	21	18.1	0.02
Depression	16	13.8	21	18.1	0.11
Anxiety	14	12.1	13	11.2	0.03
Chronic Pain	7	6.0	7	6.0	0
*Surgical Characteristics*					
Breast Mass Size (largest dimension in cm), median [quartile]	8 [5, 16.3]		11 [6.8, 17]		0.07
Breast Mass Located Medial to Nipple	25	21.6	29	25.0	0.07
Bilateral Surgery	1	0.9	2	1.7	0.07
General Anesthesia (versus Monitored Anesthesia Care)	106	91.4	106	91.4	0

Propensity-score matching was based on a nearest neighbor approach using the logit for each confounder to utilization of serratus block (without replacement). An SMD of < 0.2 was considered adequately matched

*Abbreviations*: *ASA* American Society of Anesthesiologists, *SMD* absolute standardized mean difference

**Fig. 1:**
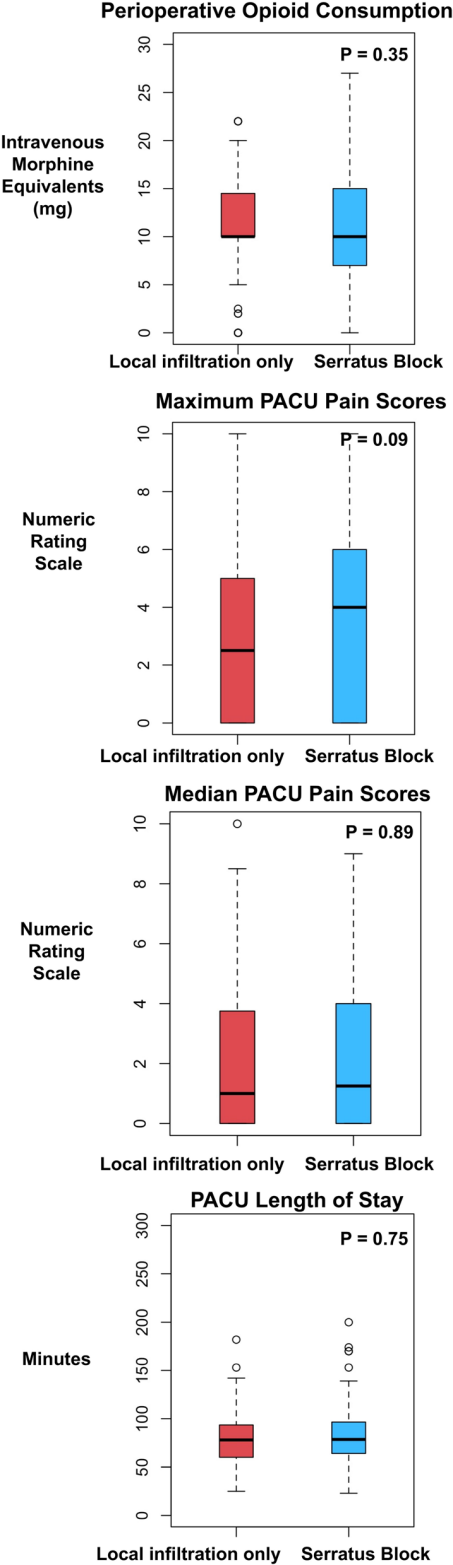
Box plots illustrating differences between propensity-score matched cohorts (surgical site infiltration only versus SAP blocks) in perioperative opioid consumption (measured in intravenous morphine equivalents consumed intraoperatively and in the PACU), median PACU pain scores (numeric rating scale 0–10), maximum PACU pain scores (numeric rating scale 0–10), and PACU length of stay (minutes). Wilcoxon signed rank test was used to measure statistically significant differences between matched cohorts. Abbreviations: PACU, post-anesthesia care unit

The median [quartile] perioperative opioid consumption in the surgical site infiltration only versus SAP block cohorts were 10 mg [10, 14.5 mg] and 10 mg [7, 15 mg], respectively (*P* = 0.35). The median [quartile] PACU pain score on the NRS scale in the surgical site infiltration only versus SAP block cohorts were 1 [0, 3.7] and 1.25 [0, 4], respectively (*P* = 0.89). The median [quartile] maximum PACU pain score on the NRS scale in the surgical site infiltration only versus SAP block cohorts were 2.5 [0, 5] and 4 [0, 6], respectively (*P* = 0.09). The median [quartile] PACU length of stay in the surgical site infiltration only versus SAP block cohorts were 78 minutes [60, 93.3 minutes] and 78.5 minutes [64, 96.25 minutes], respectively (*P* = 0.75) (Fig. 1).

## Discussion

To our knowledge, this is the first study to investigate the potential benefit of regional anesthesia for outpatient simple lumpectomies without lymph node dissection. Although this was a retrospective study, this design allows for the inclusion of a much larger sample size compared to previous studies looking at SAP versus no regional anesthesia intervention. Furthermore, both cohorts were propensity matched controlling for a variety of factors associated with increased risk of perioperative pain such as age, BMI, and chronic pain [2, 4, 6]. There was no significant difference between surgical site infiltration only versus SAP block cohorts with respect to opioid consumption, pain scores, and PACU length of stay. Based on these findings, it may not be recommended to routinely perform SAP blocks for all patients undergoing simple lumpectomies who already will receive surgical site infiltration with local anesthesia.

While PVB has been established to be effective to provide superior and excellent analgesia for breast surgery [18], its use is often limited to more invasive breast surgery due to higher technical difficulty and increased risk of neuraxial spread and pneumothorax. Some randomized controlled trials have shown limited analgesic benefit of SAP compared with no regional anesthesia block after a variety of breast surgery procedures [19, 20], none exclusively address lumpectomy only patients. Our findings are also similar to those of Abdallah et al. who reported no difference in pain scores or opioid requirement in the setting of protocolized analgesic regimen when comparing SAP vs sham after simple or partial mastectomy with sentinel node biopsy [15]. However, one important difference and an additional result from our study is that the pain from a simple lumpectomy is minimal and thus little effect can be seen between surgical site infiltration only versus SAP block with as median PACU pain score are 2 and 1.25, respectively. These data suggest that performing a SAP block for simple lumpectomies without axillary involvement should not necessarily be routine practice.

As mentioned above, SAP blocks are mostly indicated for breast oncologic surgeries with expected pain in the distribution of the lateral cutaneous branches of the intercostal nerves, which is typically the lateral half of the breast [21]. While the exact distribution of the serratus anterior block may vary based on specific technique, it is unlikely that patients with a lumpectomy performed on the medial aspect of their breast would have any benefit from the SAP block. Importantly, our study has differentiated the position of the lumpectomy and found no clinical benefit for patients with either medial or lateral surgeries.

Limitations of this study include the retrospective nature of the study design, which limits conclusions about any relationship between regional interventions and outcomes as correlative instead of causative. Likewise, procedure documentation did not reflect superficial versus deep SAP technique and there was small variability among volume and local anesthetic concentrations used. One additional concern was the consistency of the local anesthetic that was given in the operating room by the various surgical teams. While this was not standardized, it always fell below the toxic limit and was very dilute compared to the regional block performed. Furthermore, there may be other confounders not included in our adjusted analysis that would be difficult to gather retrospectively. For definitive results, a large, appropriately powered, randomized controlled trial would need to be performed.

## Conclusions

This negative retrospective cohort study suggests that patients undergoing lumpectomies without lymph node involvement may not benefit from a preoperative SAP regional intervention when compared to local infiltration at the incision site by a surgeon. This may help limit unnecessary risks for patients moving forward.

## Data Availability

The datasets generated and/or analyzed during the current study are not publicly available due to potential patient privacy compromise but are available from the corresponding author on reasonable request.

## Abbreviations

SAP: Serratus anterior plane
PACU: Post-anesthesia care unit
PECS: Pectoral nerve block
ESP: Erector Spinae Plane
PVB: Paravertebral Block
MEQ: Morphine equivalents
NRS: Numeric rating scale

## References

[1] Fecho K, Miller NR, Merritt SA, Klauber-Demore N, Hultman CS, Blau WS (2009). Acute and persistent postoperative pain after breast surgery. Pain Med.

[2] Habib AS, Kertai MD, Cooter M, Greenup RA, Hwang S (2019). Risk factors for severe acute pain and persistent pain after surgery for breast cancer: a prospective observational study. Reg Anesth Pain Med.

[3] Mejdahl MK, Andersen KG, Gärtner R, Kroman N, Kehlet H (2013). Persistent pain and sensory disturbances after treatment for breast cancer: six year nationwide follow-up study. Bmj..

[4] Schreiber KL, Zinboonyahgoon N, Xu X, Spivey T, King T, Dominici L (2019). Preoperative psychosocial and psychophysical phenotypes as predictors of acute pain outcomes after breast surgery. J Pain.

[5] Wang L, Cohen JC, Devasenapathy N, Hong BY, Kheyson S, Lu D (2020). Prevalence and intensity of persistent post-surgical pain following breast cancer surgery: a systematic review and meta-analysis of observational studies. Br J Anaesth.

[6] Wang L, Guyatt GH, Kennedy SA, Romerosa B, Kwon HY, Kaushal A (2016). Predictors of persistent pain after breast cancer surgery: a systematic review and meta-analysis of observational studies. Cmaj..

[7] Spivey TL, Gutowski ED, Zinboonyahgoon N, King TA, Dominici L, Edwards RR (2018). Chronic pain after breast surgery: a prospective, Observational Study. Ann Surg Oncol.

[8] Ilfeld BM, Madison SJ, Suresh PJ, Sandhu NS, Kormylo NJ, Malhotra N (2015). Persistent postmastectomy pain and pain-related physical and emotional functioning with and without a continuous paravertebral nerve block: a prospective 1-year follow-up assessment of a randomized, triple-masked, placebo-controlled study. Ann Surg Oncol.

[9] Desai M, Narayanan MK, Venkataraju A (2020). Pneumothorax following serratus anterior plane block. Anaesth Rep.

[10] Cheng GS, Ilfeld BM (2017). An evidence-based review of the efficacy of perioperative analgesic techniques for breast Cancer-related surgery. Pain Med.

[11] Blanco R, Parras T, McDonnell JG, Prats-Galino A (2013). Serratus plane block: a novel ultrasound-guided thoracic wall nerve block. Anaesthesia..

[12] Mayes J, Davison E, Panahi P, Patten D, Eljelani F, Womack J (2016). An anatomical evaluation of the serratus anterior plane block. Anaesthesia..

[13] Gabriel RA, Swisher MW, Sztain JF, Curran BP, Said ET, Abramson WB (2021). Serratus anterior plane versus paravertebral nerve blocks for postoperative analgesia after non-mastectomy breast surgery: a randomized controlled non-inferiority trial. Reg Anesth Pain Med.

[14] Altıparmak B, Korkmaz Toker M, Uysal A, Turan M, Gümüş DS (2019). Comparison of the effects of modified pectoral nerve block and erector spinae plane block on postoperative opioid consumption and pain scores of patients after radical mastectomy surgery: a prospective, randomized, controlled trial. J Clin Anesth.

[15] Abdallah FW, Patel V, Madjdpour C, Cil T, Brull R (2021). Quality of recovery scores in deep serratus anterior plane block vs. sham block in ambulatory breast cancer surgery: a randomised controlled trial. Anaesthesia..

[16] Swisher MW, Wallace AM, Sztain JF, Said ET, Khatibi B, Abanobi M (2020). Erector spinae plane versus paravertebral nerve blocks for postoperative analgesia after breast surgery: a randomized clinical trial. Reg Anesth Pain Med.

[17] Abdallah FW, MacLean D, Madjdpour C, Cil T, Bhatia A, Brull R (2017). Pectoralis and serratus fascial plane blocks each provide early analgesic benefits following ambulatory breast Cancer surgery: a retrospective propensity-matched cohort study. Anesth Analg.

[18] Jacobs A, Lemoine A, Joshi GP, Van de Velde M, Bonnet F, collaborators PWG. (2020). PROSPECT guideline for oncological breast surgery: a systematic review and procedure-specific postoperative pain management recommendations. Anaesthesia..

[19] Yao Y, Li J, Hu H, Xu T, Chen Y (2019). Ultrasound-guided serratus plane block enhances pain relief and quality of recovery after breast cancer surgery: a randomised controlled trial. Eur J Anaesthesiol.

[20] Mazzinari G, Rovira L, Casasempere A, Ortega J, Cort L, Esparza-Minana JM (2019). Interfascial block at the serratus muscle plane versus conventional analgesia in breast surgery: a randomized controlled trial. Reg Anesth Pain Med.

[21] Costa F, Strumia A, Remore LM, Pascarella G, Del Buono R, Tedesco M (2020). Breast surgery analgesia: another perspective for PROSPECT guidelines. Anaesthesia..

